# Parental Reflective Functioning and Its Association With Parenting Behaviors in Infancy and Early Childhood: A Systematic Review

**DOI:** 10.3389/fpsyg.2022.765312

**Published:** 2022-03-03

**Authors:** Lydia Yao Stuhrmann, Ariane Göbel, Carola Bindt, Susanne Mudra

**Affiliations:** Department of Child and Adolescent Psychiatry, Psychotherapy and Psychosomatics, University Medical Center Hamburg-Eppendorf, Hamburg, Germany

**Keywords:** parental reflective functioning, parental mentalization, parenting, parent–child interaction, infancy, early childhood, systematic review

## Abstract

**Background:**

Parental reflective functioning (PRF) refers to parents’ mental capacity to understand their own and their children’s behaviors in terms of envisioned mental states. As part of a broader concept of parental mentalization, PRF has been identified as one of the central predictors for sensitive parenting. However, the unique contribution of PRF to the quality of various parenting behaviors has not yet been addressed systematically. Thus, the present article provides a systematic overview of current research on the associations between PRF or its sub-dimensions and observed parenting behaviors in infancy and early childhood, while considering the influence of contextual factors.

**Methods:**

The review was conducted following the Preferred Reporting Items for Systematic Review and Meta-Analysis (PRISMA) guidelines. Systematic searches were carried out in five electronic databases. The eligibility and methodological quality of the identified studies were assessed using pre-defined criteria and a standardized checklist.

**Results:**

Sixteen studies with moderate to high quality on a total of 15 parenting behaviors were included, the majority of which examined positive parenting behaviors, while negative parenting behaviors were rarely investigated. Most of the associations indicated a positive effect of PRF on parenting behavior, with mostly small-sized effects. The strength and direction of the associations varied depending on the dimensionality of PRF, observation settings, sample types, socioeconomic factors, and cultural background. Moreover, five assessment instruments for PRF and 10 observation instruments for parenting behaviors were identified.

**Conclusion:**

In summary, PRF has shown a positive association with parenting quality. However, its complex interaction with further contextual factors emphasizes the need for differentiation of PRF dimensions and the consideration of the observation settings, assessment time points, psychosocial risks, and sample types in observational as well as intervention studies. Further high-quality studies with multivariate analyses and diverse study settings are required.

## Introduction

During infancy and early childhood, parental mentalization and parenting behavior are central to the quality of attachment relationships and child development (Fonagy and Target, 1997; Zeegers et al., 2017). Parental mentalization is a parent’s ability to treat the child as a psychological agent and represents an umbrella concept under which *reflective functioning* (RF) is one of the most prominent and defining constructs (Fonagy et al., 1991). Theoretically, parents’ RF is a psychological process that underlies parental self-regulation and co-regulation of the child’s affective states. In this context, parenting behavior can be seen as a subsequent expression of parents’ RF as well as a crucial pathway in the transmission of parental emotion regulation to child’s affect regulation, as illustrated by the process of marked affect-mirroring (Fonagy et al., 2002; Slade et al., 2005). In the present review, we focus on a specific form of RF in parents, namely *parental reflective functioning* (PRF), and systematically summarize associations between PRF and parenting behaviors.

### Defining PRF

The original concept of RF is related to adults’ early relationship with their caregivers and is commonly measured *via* RF coding using the Adult Attachment Interview (George et al., 1984; Fonagy et al., 1998). For differentiation, we refer to it as *adult RF* hereafter. On the contrary, the later introduced PRF focuses on parents’ RF capacity to reflect on their own and their children’s experiences in the current parent–child relationship. Specifically, PRF describes parents’ mental capacity to understand their own and their children’s behaviors in light of plausible underlying mental states (Slade, 2005), thus capturing the reflective component of parental attachment-related mental representation.

Both forms of RF in parents have been linked not only with parent–child attachment security but also with further aspects of child social–emotional development such as mentalizing abilities, emotion regulation, adolescent adjustment, and mental health (Benbassat and Priel, 2012; Esbjørn et al., 2013; Borelli et al., 2016; Ensink et al., 2016a; Nijssens et al., 2020; Bianco et al., 2021).

Although many empirical studies have investigated RF in parents, the concepts of adult RF and PRF often remain undifferentiated (Camoirano, 2017). However, differentiation is especially relevant since mentalization is, to some degree, relationship-specific (Luyten et al., 2017b). In other words, reflection on past experiences with one’s own caregivers could differ from reflection on a developing relationship with the own child considerably. Additionally, PRF could also be influenced by child characteristics such as temperament and further develop due to the interactive nature of the parent–child relationship (Sharp and Fonagy, 2008). In particular, although both forms of RF overlap, PRF taps into the parent’s reflective process underlying the current relationship with the child more directly (Slade, 2005).

The central issue related to the understanding of PRF is its operationalization because the construct is multidimensional and involves implicit and explicit mental processes regarding both cognition and affect (Luyten et al., 2019). Accordingly, the empirically revealed sub-dimensions of PRF vary depending on assessment methods (e.g., Smaling et al., 2016b; Luyten et al., 2017b). Specifically, there is a significant difference between the interview and questionnaire measures. The most commonly used method to assess PRF is the Parent Development Interview-Revised (PDI-R; Slade et al., 2004a), a semi-structured interview that asks about parents’ experiences in their parental role, perception of the child and relationship to the child, as well as experience with their own parents. The standard coding procedure is the adapted RF coding for the PDI-R (PDI-RF; Slade et al., 2004b), based on verbatim transcripts of the interview. The standard overall score of PDI-RF reflects the parent’s typical level of PRF capacity, indicating to what degree the parent could generally process multiple perspectives and explicitly reflect on the complex interactions between mental states and behaviors using specific daily situations with the child. The PDI-RF coding can also be applied to other interviews to assess PRF.

Another frequently used alternative is the Parental Reflective Functioning Questionnaire (PRFQ; Luyten et al., 2017a). The questionnaire-based self-report measure of RF involves methodological difficulty because “individuals need to rely on their capacity for mentalizing in responding to questions about mentalizing” (Fonagy et al., 2016). Thus, parents must take a meta-perspective to appraise their own mental states based on pre-selected statements. In contrast, in interviews, parents are required to mentalize about specific daily situations freely and are less able to control or appraise their narrative, on which the coding is based. Consequently, the two forms of operationalization would assess different aspects of PRF.

Besides PRF, two other concepts are also considered central under parental mentalization: parental mind-mindedness and parental insightfulness, both developed to capture parents’ ability to see things from the child’s perspective as the core of parental sensitivity and are primarily measured with a behavioral component using parent–child interaction (Meins et al., 2001; Oppenheim and Koren-Karie, 2013). Thus, although overlapping in their focus on parental capacity to see the child as a thinking and feeling individual, the concepts measure different aspects of parental mentalizing ability (Zeegers et al., 2017). PRF captures complex mental reflection not directly linked to parental behavior, whereas the other two concepts focus mainly on the mental component of parental behavioral competence. Hence, it is essential to focus on the specific concepts separately to better understand the role of parental mentalizing ability in association with parenting outcomes.

### PRF in the Context of Early Parenting

PRF has been conceptualized as a mental capacity connected to parents’ ability to co-regulate their child’s affective states in the early parent–child interaction (Slade, 2005), which provides the primary social context for children. In other words, a parent’s capacity to make sense of the child’s behaviors and internal experiences (e.g., distress, fear) is closely linked with the parental ability to respond to the child accordingly through affectively attuned parenting behavior (Grienenberger et al., 2005). Particularly in infancy and early childhood, interactions are characterized by high dependency, intense emotionality, and rapid developmental changes that require more differentiated parental reflective capacity and behavioral adaptation (Tronick, 2017; Ensink et al., 2019). The experience of being seen and treated as a thinking and feeling individual with their own mental states can, in turn, help infants integrate and then, later on, regulate their own mental states through the process of internalization (Fonagy et al., 2002). Also, it is important to consider that children can influence or react to parenting individually (Tronick and Beeghly, 2011; Slagt et al., 2016; Belsky and van IJzendoorn, 2017). However, to keep our research question specific, the present review only focuses on parental behavior in association with PRF without the additional variance from the child’s perspective.

Various parental behaviors have been associated empirically with PRF, such as disrupted affective communication, sensitivity, tolerance of infant distress, and emotion language use (Grienenberger et al., 2005; Borelli et al., 2012; Rutherford et al., 2013; Ensink et al., 2016b). As part of a narrative review, Camoirano (2017) reported a positive association between adult RF as well as PRF and the quality of caregiving and stressed the relevance of differentiating the two forms of RF. Additionally, the examined behaviors summarized under the quality of caregiving were considerably heterogeneous. In a meta-analysis, Zeegers et al. (2017) provided valuable evidence identifying the umbrella concept of parental mentalization as a predictor for parental sensitivity. Nonetheless, PRF was grouped with parental mind-mindedness and insightfulness, with only three of the 14 studies on the association between parental mentalization and sensitivity assessing PRF. Further, the included samples showed high homogeneity (i.e., mainly Western community samples), partially due to the meta-analytic approach.

Although the association between parental mentalization or parents’ RF and the parenting quality appears to be clear, a recent meta-analysis on interventions for PRF improvement found no evidence for a significant improvement in parent–child interaction, partially due to the heterogeneity of behavioral measures (Barlow et al., 2020). The authors further noted that the behavioral improvement might not be merely grounded in an increase of sensitive parenting but a reduction of disruptive parenting. In line with this, Bernier et al. (2014) stressed that a broadened view of parental behavior is necessary to understand early attachment relationships better.

Depending on the specific research context, parenting behaviors can be measured using highly heterogeneous instruments but ultimately categorized into positive (e.g., sensitive, warm, affectionate, and supportive) and negative (e.g., insensitive, disruptive, controlling, and unresponsive). The generic categories of positive and negative parenting were previously identified empirically as distinct constructs with different determinants and influences on offspring outcomes (Belsky, 1984; Simons et al., 1990; Dallaire et al., 2006). Thus, they do not represent two poles of the same dimension and are not mutually exclusive. Positive parenting has been previously linked with parental competence, whereas negative parenting with more stressors (Thomson et al., 2014).

Consequently, it can be assumed that PRF does not impact positive and negative parenting equally. As an essential part of emotion regulation (Fonagy et al., 2002), parents with higher PRF could be more resistant to emotional distress by regulating the child’s affects and their own heightened emotions and, therefore, avoid negative reactions to the child’s needs instead of acting out on impulse. On the positive side, PRF is considered the basis for parents to understand the child’s internal states underlying behavior (Slade, 2005), which could promote their ability to better tune in to the child’s affects by showing, for example, sensitive reactions. As Barlow et al. (2020) argued, promoting PRF might not directly help parents to adapt more sensitive and responsive behavior but rather to recognize and prevent dysfunctional behavior, particularly relevant for high-risk samples (Madigan et al., 2006).

Overall, the specific contribution of PRF and its sub-dimensions in the context of early parenting is still not fully understood. Moreover, it is also relevant to consider the variability of methodological factors in the study settings and further contextual factors regarding the broader social and cultural environment that potentially influence both parental mentalizing ability (Lee et al., 2020; Sleed et al., 2020) and parenting practice (Kotchick and Forehand, 2002). Since PRF has often been investigated in high-risk samples (Sleed et al., 2020), it is presumably essential to consider the influence of such contextual factors. However, there is no previous overview of parenting behaviors associated with PRF and whether the associations differ depending on other contextual factors. A qualitative synthesis in the form of a systematic review allows these differentiations.

### The Present Review

Increasing evidence has linked parental mentalization with parenting quality. However, the concepts under parental mentalization measure diverse aspects of parental mentalizing ability and capture different processes related to parents’ mental representation and behavioral competence. Although PRF has often been discussed and studied in the context of early parenting, to the best of our knowledge, no systematic review about the specific association between PRF and parenting behaviors exists thus far. Moreover, we draw attention to methodological issues related to the assessment (e.g., PRF operationalization, study settings) and sociocultural factors, potentially leading to variations in the resulting associations. Identifying these potential differences and related factors would help future research to apply methods in a more targeted manner depending on specific research questions.

To this end, the present review provides a systematic overview of empirical studies on PRF and its association with parenting behaviors in infancy and early childhood (0–5 years of age). For a better methodological orientation, two research questions regarding assessment will be addressed: (1) Which instruments have been used to assess PRF? (2) Which parenting behaviors have been examined in association with PRF? Subsequently, two main research questions will be addressed: (3) How are the associations between PRF along with its sub-dimensions and parenting behaviors? (4) Do the strengths and directions of associations between PRF and parenting behaviors vary depending on other contextual factors?

## Materials and Methods

The Preferred Reporting Items for Systematic Review and Meta-Analysis (PRISMA) guidelines (Moher et al., 2009; Shamseer et al., 2015) were followed for conducting this review. The protocol for this systematic review was registered in the PROSPERO database (registration number: CRD42019137484).

### Literature Search and Eligibility Criteria

We searched the following electronic bibliographic databases in November 2018: CINAHL, PsycINFO, PSYNDEX, PubMed/MEDLINE, and Web of Science. The search was updated in June 2021. Furthermore, we screened reference lists of eligible studies and review articles. Google Scholar was also searched. We only included the term “parental reflective functioning” and its variations in the search strategy to find all relevant references since parenting behaviors could be labeled differently. The searches were performed using the following search strategy: (parent* OR maternal OR paternal) AND (mentaliz* OR mentalis* OR reflective function*). The search strategy has been adapted for the syntax of each bibliographic database and additional German terms in the German-speaking database PSYNDEX. There were no restrictions regarding language and publication period.

We included empirical studies that (a) were published as peer-reviewed journal articles, doctoral dissertations, or published master’s theses, (b) assessed PRF referring to one specific parent–child relationship postnatally, (c) assessed parenting behavior using objective observations of parent–child interaction, and (d) reported statistical associations between PRF and observed parental behavior. Regarding the first criterion, we included gray literature sources (e.g., doctoral dissertations) paired with a quality assessment to avoid publication bias, as recommended by Cochrane (Higgins et al., 2019). Regarding the last criterion, intervention studies should report either (e) an association between the target variables both assessed pre-intervention, or (f) an association between changes in both target variables from pre- to post-intervention. We did not include self-report data of parental behavior, which might be influenced by parental perception (Herbers et al., 2017) and thus also by PRF.

Studies were excluded if (a) PRF was measured using instruments for the assessment of adult RF (e.g., RF coding on the Adult Attachment Interview), (b) parenting behavior cannot be separated from child behavior (e.g., synchrony), or (c) the sample contains children that are older than 5 years at the assessments of PRF or parenting behavior. We limited the age range of index children to keep the development-specific implications of our study focused on parent–child relationships in infancy and early childhood.

### Study Selection, Data Extraction and Synthesis

Studies identified from the literature search were screened in two steps. First, the titles and abstracts were screened to identify potentially relevant studies. Second, full texts of the studies identified in the first step were evaluated by two independent reviewers (LYS and AG) based on the eligibility criteria. The inter-rater agreement was excellent (*κ* = 0.80) according to Cicchetti and Sparrow (1981). Disagreements between the two reviewers were resolved through discussion until a consensus was reached.

For the data extraction, a pilot-tested, standardized spreadsheet was used with pre-defined variables: authors and year of publication, research question, study design and sample, PRF instrument and administration, parenting behavior instrument and administration, descriptive statistics for PRF and parenting behaviors, statistics for associations between PRF and parenting behaviors. Variables on sample characteristics included sample type (e.g., mother or father, high-risk or community), setting (e.g., location, recruitment), and parents’ and children’s age. Three authors of the included studies were contacted to request relevant data that were missing. One of the authors replied.

The extracted data were summarized based on PRF dimensions. The following aspects were considered: statistical analysis (bivariate or multivariate), statistical significance of the targeted association (significant or non-significant), parenting behaviors (positive or negative), observation setting (unstructured or structured task), and sample type (at-/high-risk or community). Statistical findings on the association between PRF and parenting behaviors were reviewed systematically to identify contextual factors linked with the association within or across the studies, that is, whether a contextual factor statistically significantly impacted the targeted association (e.g., moderating or mediating effect) or whether the targeted association differed between studies depending on the contextual factor (e.g., comparison between high-risk and community sample). Contextual factors refer to methodological (e.g., observation setting, sample types) or sociocultural factors (e.g., socioeconomic status or cultural background) that offer the context in which the association between PRF and parenting behaviors is embedded.

### Quality Assessment

The methodological quality of the included studies was assessed using an adapted checklist based on the Effective Public Health Practice Project (EPHPP) checklist (Thomas et al., 2004). Because some of the items from the EPHPP checklist are not relevant for observational studies, we additionally considered criteria from the National Institute for Health and Clinical Excellence (NICE) public health guidance and the Cochrane Collaboration Risk of Bias Tool (National Institute for Health and Care Excellence, 2012; Higgins et al., 2019). The adapted checklist assesses the following components: sample representativity, study design, data collection method, withdrawals and dropouts, and quantitative analyses. A global rating was assigned to each study depending on the ratings of the single components. We further applied ratings on three types of risk of bias for a comprehensive overview of the study quality: selection bias, detection bias, and attrition bias. Detection bias was appraised on the outcome level, while the remaining aspects were rated on the study level. The adapted checklist is available in the Supplementary Material. Two reviewers (LYS and AG) double rated 57% of the studies independently. The inter-rater agreement was again excellent (*κ* = 0.79) according to Cicchetti and Sparrow (1981). Disagreements were resolved through discussion.

## Results

**Figure 1:**
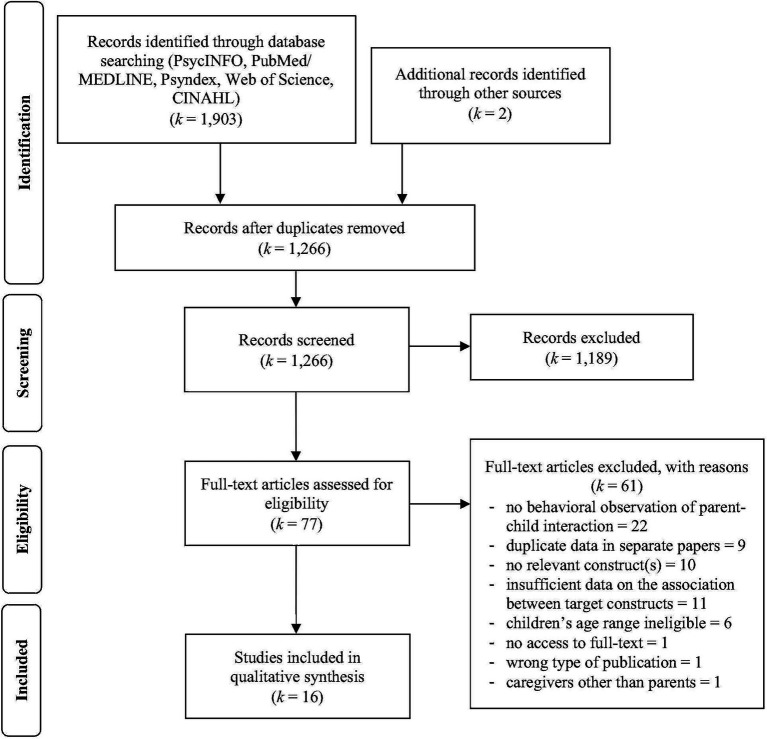
Preferred Reporting Items for Systematic Review and Meta-Analysis (PRISMA) flow diagram of the systematic review process.

Figure 1 shows the process of study identification, selection, and review. The systematic literature searches generated 1,266 references. After screening 77 full-text articles, 16 studies were included in the final review.

Among all included studies, 14 were peer-reviewed articles, one was a doctoral dissertation, and one was a published master’s thesis. Except for two studies from South Africa and Israel, all remaining studies were conducted in Western countries – Australia, Austria, Canada, Germany, Switzerland, the United Kingdom, and the United States. More specifically, almost half of the studies (*k* = 7) were from the United States. Sample sizes ranged from 26 to 163, with 44% of the sample sizes under 51, 44% between 51 and 100, and 13% over 100. Overall, *N* = 1,076 parent–child dyads were involved. While most studies focused on mothers, two studies were conducted with fathers (*n* = 148 in total). Nine of the included studies consisted of at-risk or high-risk samples, including mothers with substance abuse, interpersonal violence-related posttraumatic stress disorder (IPV-PTSD), postpartum depression, features of borderline personality disorder (BPD), and mothers in prison. One study oversampled for women with childhood maltreatment, while one study included women with pregnancy risks. The remaining five studies were conducted with community samples without any specific risks. The involved children were aged up to 60 months. Specifically, only two studies included children over 36 months old. Details of the included studies are shown in Supplementary Table 1.

Regarding the quality assessment rating, nine studies were of high methodological quality, while the remaining seven were of moderate quality. The most mixed ratings were on attrition bias followed by selection bias because one-third of the studies had either (a) a moderate rate of dropout or missing data, or (b) an incomplete report of this information, and most of the studies had relatively small sample sizes. These sample sizes and attrition rates are common in this research field, mainly using time-consuming measures and longitudinal study designs. For detailed ratings, see Table 1.

**Table 1:** Methodological quality rating (risk of biases and overall quality) of the included studies.

First author, year	Selection bias	Detection bias	Attrition bias	Overall quality
Buttitta et al., 2019	M	L	L	H
Dawson et al., 2018	M	L	L	H
Dollberg, 2021	M	L	M	H
Dunckel, 2003	H	M	L	M
Ensink et al., 2019	L	L	M	H
Grienenberger et al., 2005	H	L	M	M
Hasselbeck, 2015	M	L	H	M
Huth-Bocks et al., 2014	L	H	L	M
Krink et al., 2018	M	H	L	M
Newman-Morris et al., 2020	M	L	L	H
Perry et al., 2015	H	L	M	M
Schechter et al., 2008	M	M	L	H
Sleed et al., 2013	M	M	H	M
Suardi et al., 2020	M	L	M	H
Suchman et al., 2010	M	L	L	H
Suchman et al., 2018	L	L	L	H

L, low; M, moderate; and H, high.

### Assessment Instruments for PRF

Table 2 shows detailed information on the five instruments used to assess PRF in the included studies.

**Table 2:** Assessment instruments for parental reflective functioning (PRF) used in the included studies.

Instrument	Dimensions/subscales used	Scoring
PDI-R	Total Self-focused Child-focused	Addendum to the RF scoring manual for PDI-R, from −1 (anti-reflective) to 9 (exceptional RF)
Mini-PRFI	Total	
WMCI adapted	Total	
Highpoints/lowpoints interview	Total	
PRFQ	Pre-mentalizing modes (six items) Certainty about mental states (six items) Interest and curiosity in mental states (six items)	From 1 (strongly disagree) to 7 (strongly agree), subscale sum scores

Only dimensions/subscales used in the included studies are listed in this table. PDI-R, Parent Development Interview-Revised; RF, reflective functioning; Mini-PRFI, Mini-Parent Reflective Functioning Interview; WMCI, Working Model of the Child Interview; and PRFQ, Parental Reflective Functioning Questionnaire.

Most of the studies (*k* = 11) used the PDI-RF coding on the PDI-R. Three of these studies (Suchman et al., 2010, 2018; Buttitta et al., 2019) investigated PRF sub-dimensions using a two-factor model of the PDI-RF: self-focused and child-focused PRF. The self-focused dimension consists mainly of questions regarding a parent’s emotional experience of parenting (e.g., “How has having your child changed you?”), while the child-focused dimension consists of questions primarily regarding the child’s mental states (e.g., “Has your child ever felt rejected?”). The latter dimension also contains questions regarding the dynamics in the mental processes of parent and child in relation to each other (e.g., “Tell me about a recent time when you and your child really clicked.”). One study slightly adapted the composition of the child-focused PRF (Buttitta et al., 2019).

Additionally, the PDI-RF coding was applied for an adapted version of the Working Model of the Child Interview (WMCI; Zeanah and Benoit, 1995) in two studies and a self-developed Highpoints/Lowpoints Interview in one study. Both interviews were not established instruments for assessing PRF so that their validity remained questionable (Dunckel, 2003; Schechter et al., 2008).

Furthermore, one study developed the Mini-Parent Reflective Functioning Interview (Mini-PRFI; Ensink et al., 2019), referring more strongly to a specific parent–child interaction situation prior to the interview and focusing more on the child’s temperament. The PDI-RF coding was also applied for this interview. An average admission duration of only 15 min makes the application of this instrument very time-saving.

Lastly, one included study (Krink et al., 2018) used the self-report PRFQ with three subscales. *Pre-mentalizing modes* capture a non-mentalizing stance that reflects a parent’s inability to reflect on the child’s mental states (e.g., “Often, my child’s behavior is too confusing to bother figuring out.”). *Certainty about mental states* captures a parent’s ability to recognize the opaque nature of mental states (e.g., “I always know what my child wants.”). *Interest and curiosity in mental states* capture a parent’s active interest in understanding the child’s mental states (e.g., “I wonder a lot about what my child is thinking and feeling.”). A particularly high or low response on the certainty or the interest scales indicates nonoptimal mentalizing (e.g., overinterpreting or lack of interest in the child’s mental states). Accordingly, the scale scores are recoded to indicate that a highly reflective parent would be interested in but not too certain about mental states and show a low level of non-mentalizing stance.

### Observation Instruments for Parenting Behaviors

Of all included studies, five focused solely on sensitivity, while one study focused on insensitivity, five on multiple behaviors, two on disrupted affective communication, one on positive engagement, and one on positive as well as hostile/intrusive parenting.

We identified 10 instruments for the observation of parenting behaviors. Some of the instruments assess both parent and child behavior. Further, not all subscales of the respective instruments were used. For a better overview, only the subscales regarding parental behavior used in the included studies are described in the following. Table 3 shows detailed information on these instruments.

**Table 3:** Observation instruments used in the included studies.

Instrument	Constructs/subscales used	Scoring
Ainsworth sensitivity scale	Sensitivity	From 1 (highly insensitive) to 9 (highly sensitive)
AMBIANCE	Overall level of disrupted communication	From 1 (high normal) to 7 (disrupted communication with few or no ameliorating behaviors)
CARE-Index toddler version	Sensitivity Controlling behavior Unresponsive behavior	Scores range from 0 (insensitive) to 14 (outstandingly sensitive)
CIB	Parent positive engagement (five items) Maternal sensitivity (12 items)	Five-point scale for frequency and intensity from 1 to 5, sum scores of the respective items
DIP	Insensitivity Disconnected parenting behavior Extreme parental insensitivity	Nine-point scale from 1 to 9 each time insensitive behaviors occur; total score by averaging two subscale scores
EA Scales infancy/early childhood version 3rd and 4th edition	Sensitivity Structuring Non-intrusiveness Non-hostility	Seven-point Likert scale from 1 (low EA) to 7 (high EA)
MBQS-mini/Mni-MBQS-V	Sensitivity	Correlation between the descriptive sort and a criterion sort of a prototypically sensitive mother: *r* = −1.0 (least sensitive) to 1.0 prototypically sensitive
MIPCS	Positive parenting Behavioral sensitivity Engagement Flexibility Warmth Affective sensitivity Positive affect Negative parenting Overcontrolling/Intrusiveness Hostility	Five-point Likert scales, scores for each of the two constructs by averaging respective subscale scores
NCAST Teaching Scale	Sensitivity to cues (11 items) Response to distress (11 items) Social–emotional growth fostering (11 items) Cognitive growth fostering (17 items) Contingency rating for each of the subscales	Binary items on occurrence and non-occurrence of specific behaviors rated by 0 (no) and 1 (yes), subscale sum scores and contingency scores
PCIS	Quality of interaction (defined as maternal behavioral sensitivity) Quality Appropriateness General impression of the interaction	Five-point scale from 1 to 5, composite score by averaging subscale scores

*Only constructs/subscales used in the included studies are listed in this table. AMBIANCE, Atypical Maternal Behavioral Instrument for Assessment and Classification; CARE-Index, Child-Adult Relationship Experimental Index; CIB, Coding Interactive Behavior; DIP, Disconnected and Extremely Insensitive Parenting; EA, Emotional Availability; MBQS-mini, Maternal Behavior Q-sort mini; Mini-MBQS-V, Mini Maternal Behavior Q-sort revised; MICS, Mother Infant Coding System; MIPCS, MACY Infant–Parent Coding System; NCAST, Nursing Child Assessment Satellite Training; and PCIS, Parent/Caregiver Involvement Scale.*

The Emotional Availability Scales (EA Scales; Biringen et al., 2000; Biringen, 2008) and the Coding Interactive Behavior (CIB; Feldman, 1998) were used in three studies, while the Atypical Maternal Behavioral Instrument for Assessment and Classification (AMBIANCE; Bronfman et al., 1999), the Nursing Child Assessment Satellite Training (NCAST) Teaching Scale (Barnard and Eyris, 1979), and the Maternal Behavior Q-sort mini (MBQS-mini and Mini-MBQS-V; Moran, 2009; Pederson et al., 2009) were used in two studies, respectively. In one included study, Sleed et al. (2013) generated their own subscales of the CIB due to insufficient internal consistency of the original subscales in their sample.

The remaining instruments have been used in one of the included studies, respectively: the original sensitivity scale by Ainsworth et al. (1974), the Child-Adult Relationship Experimental Index (CARE-Index; Crittenden, 2006), the Parent/Caregiver Involvement Scale (PCIS; Farran et al., 1986), the Disconnected and Extremely Insensitive Parenting (DIP) scale (Out et al., 2009), and the Maternal Anxiety during the Childbearing Years (MACY) Infant-Parent Coding System (MIPCS; Huth-Bocks et al., 2014). The original sensitivity scale by Ainsworth was used in one study together with the MBQS-Mini. Part of the PCIS was used to aggregate a score for quality of interaction defined as maternal behavioral sensitivity (Dunckel, 2003). The MIPCS was developed in the context of the MACY study to evaluate parental, infant, and dyadic interactive behaviors associated with attachment formation (Huth-Bocks et al., 2014).

Most of the instruments are related to attachment theory and overlap in their conceptualizations, whereas the NCAST Teaching Scale was developed to detect children’s health and developmental problems.

The included studies used various interaction situations between parents and their children, from unstructured free play to highly structured tasks such as teaching tasks or the Still-Face-Paradigm (SFP; Tronick et al., 1978). Parents were usually asked to teach their children to perform a specific activity (e.g., stacking blocks) in teaching tasks. The SFP is structured into three separate episodes in the following order: play, still-face, and re-engagement episode. Only the NCAST Teaching Scale was consistently applied with a structured teaching situation of 5 min (Oxford and Findlay, 2013).

### Associations Between PRF and Parenting Behaviors

Overall, 11 studies investigated the overall level of PRF, while three studies examined two PRF sub-dimensions based on the PDI-RF. One study investigated three PRF dimensions based on the PRFQ. Most of the studies (*k* = 11) focused on positive parenting constructs, whereas the remaining studies (*k* = 5) examined negative parenting constructs only or additionally. In the following section, the associations with parenting behavior will be reported separately for the PRF sub-dimensions. Table 4 shows a simplified summary of the reported associations for a better overview.

**Table 4:** Simplified summary of the reported associations between parental reflective functioning (PRF) and parenting behaviors.

	PDI-RF coding						PRFQ^b^	
	Overall		Self-focused		Child-focused		Pre-mentalizing modes	
**Positive parenting**								
Sensitivity	Dawson et al., 2018	ns					Krink et al., 2018^c^	✓
	Dollberg, 2021	ns						
	Dunckel, 2003	ns						
	Newman-Morris et al., 2020	ns						
	Perry et al., 2015	ns						
	Hasselbeck, 2015	✓						
	Suardi et al., 2020	✓						
Sensitivity to cues			Suchman et al., 2010	✓	Suchman et al., 2010	ns		
					Buttitta et al., 2019	✓		
Social–emotional growth fostering			Suchman et al., 2010	✓	Suchman et al., 2010	ns		
					Buttitta et al., 2019	✓		
Cognitive growth fostering			Suchman et al., 2010	✓	Suchman et al., 2010	ns		
Response to distress			Suchman et al., 2010	ns	Suchman et al., 2010	ns		
Structuring	Perry et al., 2015	ns						
Non-intrusiveness	Perry et al., 2015	ns						
Non-hostility	Perry et al., 2015	ns						
Positive parenting^a^	Huth-Bocks et al., 2014	✓						
Positive engagement	Sleed et al., 2013	✓						
**Negative parenting**								
Insensitivity	Ensink et al., 2019	✓						
Disrupted affective communication	Grienenberger et al., 2005	✓						
	Schechter et al., 2008	ns						
Hostile/intrusive parenting	Huth-Bocks et al., 2014	✓						
Controlling	Suardi et al., 2020	ns						
Unresponsive	Suardi et al., 2020	ns						

ns = reported associations not significant; ✓ = at least one significant association reported. PRF, parental reflective functioning; PDI-RF coding, reflective functioning coding adapted for the Parent Development Interview-Revised (applied with various interview methods); and PRFQ, Parental Reflective Functioning Questionnaire.

a Study-defined parenting behavior.

b The PRFQ was only used in one study so that only the subscale with significant association is shown in the table due to space restrictions.

c This study also found non-significant associations between Sensitivity and the other two subscales of the PRFQ, namely Certainty about mental states, Interest and curiosity in mental states (not shown in the table due to space restrictions).

#### Associations With Overall PRF

Five out of the seven studies investigating the link between overall PRF with maternal *sensitivity* found no statistically significant association, including samples with and without psychosocial risk status, a South African at-risk sample, and an Israeli sample including pregnancy risks (Dunckel, 2003; Perry et al., 2015; Dawson et al., 2018; Newman-Morris et al., 2020; Dollberg, 2021). The remaining two studies showed partially mixed findings: Suardi et al. (2020) reported a significant medium-sized positive correlation between overall PRF and sensitivity among mothers with and without IPV-PTSD. The predictive effect of PRF on sensitivity was confirmed in a subsequent multiple regression analysis independent of IPV-PTSD. In a community sample of fathers, Hasselbeck (2015) found that the group with high PRF showed significantly higher paternal sensitivity than those with low PRF, with a large effect size. In subsequent multivariate path analysis, however, PRF showed no significant association with paternal sensitivity.

The overall PRF was not significantly correlated with maternal *structuring* and *non-intrusiveness* in two studies with psychosocial high-risk mothers and mothers without risk status (Perry et al., 2015; Newman-Morris et al., 2020). In one of the two studies, the overall PRF was significantly correlated with maternal *non-hostility*, showing a medium-sized effect (Newman-Morris et al., 2020).

A small-sized positive correlation between overall PRF and *positive engagement* was found at baseline in an intervention study with high-risk mothers (Sleed et al., 2013). However, the change in PRF from baseline to follow-up was not significantly correlated with the change in positive engagement.

The overall PRF was significantly and positively correlated with another study-defined *positive parenting* in two different interaction situations (free play and teaching) among mothers with and without childhood maltreatment, showing small- to medium-sized effects (Huth-Bocks et al., 2014). In the same study, the overall PRF was significantly negatively correlated with maternal *hostile/intrusive parenting* in only one interaction situation (free play), showing a small-sized effect. After controlling for sociodemographic factors, only a partial correlation between overall PRF and positive parenting in one interaction situation (free play) remained significant. Here, PRF was assessed at 16 months postpartum after assessing maternal behavior at 7 months postpartum. The reversed direction of assessing mental and behavioral constructs indicates that mothers who showed more positive parenting demonstrated higher PRF later on.

The association between overall PRF and maternal *disrupted affective communication* with their infants was examined in two studies. One of them (Schechter et al., 2008) found no significant association using a linear regression model in a high-risk sample. The other study (Grienenberger et al., 2005) reported a medium-sized negative correlation in a community sample, meaning mothers with higher levels of PRF had shown less disrupted behavior.

One study investigated the overall PRF and its association with maternal *insensitivity* using multiple statistical methods in a community sample (Ensink et al., 2019). Besides a small-sized negative correlation, PRF has also shown a significant negative effect on insensitivity in a hierarchical regression model, meaning a higher PRF level predicted less insensitivity. Furthermore, this effect remained significant in a regression-based mediation model, demonstrating that an increase in PRF predicted a decrease in maternal insensitivity.

Associations between overall PRF and maternal *controlling* and *unresponsive* behavior have shown to be non-significant among mothers with and without IPV-PTSD (Suardi et al., 2020).

#### Associations With Child-Focused and Self-Focused PRF

Both maternal and paternal child-focused PRF have shown no significant, independent, or direct associations with *sensitivity to child’s cues* (using a teaching task) in two studies with a high-risk and a community sample, using various statistical methods (Suchman et al., 2010; Buttitta et al., 2019). In the same two studies, child-focused PRF has also shown mostly non-significant associations with *social–emotional growth fostering*, except for one significant link in a path analysis (Buttitta et al., 2019).

Child-focused PRF was not significantly related to maternal *response to child’s distress* and *cognitive growth fostering*, using multiple regression analyses in an intervention study with a high-risk sample (Suchman et al., 2010). In the same study, self-focused PRF was not significantly associated with maternal response to child’s distress but was significantly associated with sensitivity to child’s cues, social–emotional growth fostering, and cognitive growth fostering, with small-sized effects. Taken together, the child-focused and self-focused PRF have shown almost opposite effects on maternal behaviors in this study, indicating a positive effect of self-focused PRF on maternal parenting.

Furthermore, the improvement in child-focused PRF has shown a positive, small-sized effect on the improvement in maternal *sensitivity*, whereas the improvement in self-focused PRF showed no effect in another intervention study with a high-risk sample (Suchman et al., 2018). However, it was unclear whether maternal PRF made a significant contribution independent of the improvement in maternal representation of the caregiving relationship in the statistical model.

#### Associations With PRFQ Dimensions

One study closely examined dimensions of the PRFQ and maternal *sensitivity* in an at-risk sample during the play and the re-engagement episode of the SFP separately (Krink et al., 2018). No significant correlation between the constructs could be found in each SFP episode separately, whereas the results showed a significant, small-sized correlation between pre-mentalizing modes and decreased maternal sensitivity between the two SFP episodes. Further, the certainty and the interest subscales were not significantly correlated with maternal sensitivity.

#### Summary of the Reported Associations

Overall, only a few studies have investigated the association between PRF and negative parenting behaviors. For both positive and negative parenting constructs, the effect sizes of the association with PRF and its sub-dimensions are mainly small to nearly medium. Only one study found a large-sized effect (Hasselbeck, 2015), while four studies found medium- to nearly large-sized effects, all using bivariate statistical methods (Grienenberger et al., 2005; Ensink et al., 2019; Newman-Morris et al., 2020; Suardi et al., 2020). Six studies reported associations based on both bivariate and multivariate statistical methods, of which over half of them revealed a considerable alteration of the bivariate effect (Huth-Bocks et al., 2014; Hasselbeck, 2015; Buttitta et al., 2019; Newman-Morris et al., 2020). The altered effects highlight the relevance of other influencing factors. Moreover, the child-focused and self-focused PRF have shown differentiated effects depending on the study setting.

### Contextual Factors Influencing the Associations

Among the reported associations between PRF and parenting behaviors, additional influence of the following contextual factors were analyzed in the studies: observation setting (*k* = 2), family income (*k* = 2), and cultural background (*k* = 1). Moreover, different sample types are also relevant when compared within (*k* = 2) or across (*k* = 4) the studies.

The effect of observation settings was directly shown in two studies. In the study by Krink et al. (2018), PRF was only significantly correlated with maternal sensitivity when sensitivity was measured as a difference between two SFP episodes, indicating higher levels of pre-mentalizing modes linked with a larger decrease of sensitivity. The decrease of maternal sensitivity indicated the effect of emotional distress induced by the re-engagement after a still-face situation. Hence, this finding shows a stronger association between concurrent PRF and parenting behavior under emotional distress. In contrast, Huth-Bocks et al. (2014) showed a robust correlation between overall PRF and positive parenting using free play compared to a teaching task, indicating a stronger association between preceding maternal parenting under less emotional distress and PRF measured 9 months later.

The effect of family income was highlighted in two studies. Buttitta et al. (2019) linked child-focused PRF indirectly to paternal parenting by revealing a moderating effect of child-focused PRF on the link between family income and paternal sensitivity to child’s cues. More specifically, family income was positively associated with paternal sensitivity to cues only for fathers with low child-focused PRF. This interaction between child-focused PRF and family income was not significantly linked with paternal social–emotional growth fostering (Buttitta et al., 2019). In another study, the correlation between PRF and maternal behavior was markedly reduced and turned non-significant when controlled for sociodemographic factors, including family income risk, although the effect with positive parenting was robust and partially remained significant (Huth-Bocks et al., 2014).

The effect of the cultural background was analyzed in one study from South Africa (Dawson et al., 2018). By comparing two different measurements of maternal sensitivity, the authors reported a near-significant, nearly medium-sized correlation between PRF and sensitivity assessed by Ainsworth’s original scale, while the small-sized association with sensitivity assessed by MBQS-mini was far from reaching the significance level. The difference between the two correlations was also near-significant (*z* = 1.48, *p* = 0.07) and could be grounded in the cultural implications of the assessment methods. Despite the substantial overlap between the two coding schemes, the MBQS-mini contains detailed criteria regarding culturally specific aspects of the interaction, such as verbal responsiveness, whereas Ainsworth’s original scale offers a more holistic picture of maternal sensitivity (Mesman and Emmen, 2013).

Some studies with community and high-risk samples assessed the same PRF dimensions and parenting behaviors, allowing comparisons between the sample types. Using the same behavioral assessment, Grienenberger et al. (2005) showed a nearly large-sized correlation between PRF and maternal negative parenting behavior in a community sample, whereas Schechter et al. (2008) found no significant association in a high-risk sample of mothers exposed to interpersonal violence. Mothers in the latter study showed overly low variability of PRF and maternal behavior, indicating low PRF and low parenting quality in the high-risk sample. Moreover, Buttitta et al. (2019) found positive effects of child-focused PRF on positive parenting behaviors in a community sample of fathers. In contrast, child-focused PRF showed no significant effects on the same parenting behaviors in a high-risk sample of substance-using mothers (Suchman et al., 2010). Instead, self-focused PRF had shown a positive effect on maternal behaviors in the high-risk sample.

Nonetheless, two studies directly comparing at-risk or high-risk and comparison groups showed no significant difference in the association between PRF and maternal behaviors. Perry et al. (2015) compared PRF and maternal positive parenting behaviors in a high-risk group of substance-abusing mothers and a comparison group without current substance use problems. Their results indicated no significant group differences regarding either PRF or parental behavior. Suardi et al. (2020) compared mothers with and without IPV-PTSD and also found no significant difference in the correlation between PRF and maternal positive and negative parenting behaviors. Both studies had small sample sizes that did not allow more complex statistical analyses for additional exploration.

Lastly, the two studies using paternal community samples showed relatively low levels of PRF compared to maternal samples and limited findings on its effect on paternal positive parenting behaviors (Hasselbeck, 2015; Buttitta et al., 2019).

## Discussion

This systematic review synthesized empirical studies on PRF and its association with parenting behaviors during infancy and early childhood. Besides statistical data on the strength and direction of the associations, we also summarized the assessment instruments and addressed other contextual factors that have shown a substantial influence on the associations.

Although most of the studies examined the overall PRF, three studies focused on its sub-dimensions. Further, there were various parenting behaviors, the majority of which can be categorized as positive parenting. In total, there were more results on parental sensitivity than on other behaviors. Most of the studies (*k* = 10) reported significant associations between PRF and parenting behaviors in the theoretically expected directions, with small- to medium-sized effects, using various statistical methods.

Nonetheless, the associations varied considerably depending on the PRF sub-dimensions and contextual factors, including observation settings, sample types, family income, and cultural background. Specifically, compared to lower distress conditions, there are indications that the association between PRF and parenting behaviors tends to be more robust under emotional distress as well as in more difficult life circumstances with less socioeconomic or emotional resources. Furthermore, PRF sub-dimensions assessed using PDI-RF seem to have different effects depending on sample characteristics.

### Multidimensionality of PRF in Association With Parenting Behavior

Several studies indicated that the link between PRF and parenting varied depending on the dimensionality of PRF. The sub-dimensions of PDI-RF differentiate the relational focus and contain more dynamic aspects of the relationship, whereas the PRFQ dimensions aim to measure more generic key features of PRF representing mental processes that are already considered in the PDI-RF coding (Luyten et al., 2017a).

Regarding the PDI-RF sub-dimensions, two intervention studies revealed that the predicting effect of the self-focused and child-focused PRF on parenting quality varied depending on whether the intervention effect was taken into account. Specifically, without the intervention effect, only higher *self-focused* PRF in high-risk mothers was linked with higher parenting quality, whereas only the improvement of *child-focused* PRF through mentalization-based intervention predicted a behavioral improvement (Suchman et al., 2010, 2018). Regarding the positive effect of self-focused PRF pre-intervention, Suchman et al. (2010) argued that the self-focused questions in the PDI-R refer to difficult affective experiences and are therefore emotionally more challenging than the child-focused questions. Since substance use can be understood as a dysfunctional way of emotion regulation, reflecting these questions could be a more vital and meaningful mental capacity in the association with parenting behavior among parents with substance use problems. Another related issue to these findings could be a difference in the rate of change for PRF and parenting behavior (Sleed et al., 2013). More specifically, behavioral changes may take longer to become evident than changes in PRF (Barlow et al., 2020). Accordingly, it would be necessary to adjust the interval and frequency of post-treatment follow-ups to determine whether and how the changes in both constructs are related to each other. For more frequent follow-up assessments, new instruments developed for less time-consuming PRF assessments could be helpful, such as the Mini-PRFI described earlier or the Reflective Functioning Five Minute Speech Sample that is currently being validated (Adkins et al., 2021). Moreover, the difference in the rate of change could also apply to the self-focused and child-focused dimensions of PRF, especially since self-focused PRF contains complex reflections on mothers’ own negative emotional experiences that could be particularly difficult for high-risk mothers to access.

The included study using the PRFQ (Krink et al., 2018) indicated a specific role of pre-mentalizing modes in the association with maternal sensitivity under emotional distress in their clinical sample. This finding is in line with the conceptualization of pre-mentalizing modes being characteristic for parents with RF impairments that are often associated with a variety of psychopathology (Luyten et al., 2017a). Moreover, a higher level of maternal pre-mentalizing modes has been previously linked with children’s early regulatory problems and parenting stress (Georg et al., 2018). Taken together, this indicates the specific adverse impact of the non-mentalizing stance on sensitive parenting in clinical samples with disruptions in the early mother–child relationship. These mothers might have difficulties “to enter into the subjective world of the child” (Luyten et al., 2017a) due to the present symptoms or the stress induced by the symptoms of themselves or their infants. This reflective difficulty manifested as pre-metalizing mode could become one of the crucial factors influencing maternal behavior.

It should be noted that there are methodological issues related to the factorial structures of the PDI-RF and the PRFQ. Inconsistency exists regarding the PDI-RF sub-dimensions. The two-factor model applied in our included studies was only developed in a small high-risk sample with relatively low internal consistency and did not entirely fit other samples (Borelli et al., 2016; Suchman et al., 2018; Buttitta et al., 2019). Besides the two-factor model, another three-factor model additionally contains a relation-focused dimension (Smaling et al., 2016b). Thus, the effect of the improved child-focused PRF on maternal behavioral change through the intervention mentioned above could also be understood as an improved maternal understanding of not only their infants’ internal states but also the interactional processes with their infants.

Furthermore, the focus of parental reflection during the PDI-R is not limited to the focus of the interview questions. Parents could reflect on both their own or their children’s mental states at any time throughout the interview, and the PDI-RF coding takes both aspects into account. For example, if a mother was asked whether her child ever felt rejected, she could also talk about her own feelings or thoughts related to this question without details about her child’s possible mental states. Thus, it is difficult to determine whether a higher score on the self-focused or child-focused interview question is also qualitatively connected to the parent’s reflection with the respective focus.

In the case of the PRFQ, the structural validity of several available language versions is still unknown, including the version used in the included study (Krink et al., 2018). Specifically, studies indicate that the original three-factor structure could not be consistently confirmed in some language versions (Pajulo et al., 2018; Lee et al., 2020). Additionally, the internal consistencies of the subscales were partly low or questionable in previous studies (Burkhart et al., 2017; Georg et al., 2018; Krink et al., 2018). Differences in factorial structures might imply cultural differences and shed light on further details of the associations between PRF and parenting behaviors.

### Associations With Positive and Negative Parenting

Nearly all of the parenting observation instruments in this review had a theoretical background related to attachment theory. Although many were labeled differently, most of the behaviors were directly or indirectly related to the broader concept of parental sensitivity, which has been defined and operationalized beyond the original conceptualization by Mary Ainsworth in past research (Mesman and Emmen, 2013). Since the labeling of parenting behaviors varies depending on theoretical context and operationalization, we only referred to the constructs as they were labeled in the respective instruments.

In summary, 15 parenting behaviors were examined. Most of the findings across all included studies were on parental sensitivity. Statistically significant associations were found between PRF and most behaviors, except structuring, non-intrusiveness, response to distress, controlling, and unresponsiveness.

Studies in the current review indicate that PRF and its sub-dimensions were generally positively associated with positive parenting behaviors (e.g., sensitivity, social–emotional growth fostering, and non-hostility) and negatively associated with negative parenting behaviors (e.g., disruptive affective communication, insensitivity). The reported effects were mainly small. However, negative parenting constructs were rarely examined. Only maternal insensitivity has shown the most robust significant association with PRF using multiple statistical methods (Ensink et al., 2019). Further, the two included studies examining both positive and negative behaviors demonstrated only significant links between PRF and positive parenting behaviors (Huth-Bocks et al., 2014; Suardi et al., 2020). This finding is in line with a study on prenatal PRF, in which the predictive effect of PRF has only been shown to be significant on positive parenting behavior in multivariate analyses despite the significant bivariate correlations with both positive and negative parenting behaviors (Smaling et al., 2016a). Nonetheless, most of the included studies found none or limited associations even with parental sensitivity. Although somewhat unexpected, this finding aligns with the variations in effect sizes due to inconsistent sensitivity measures described by Zeegers et al. (2017) and the “loose coupling” between parental attachment security, PRF, and parental sensitivity described by Luyten et al. (2017b), indicating influence from other related factors.

### Contextual Factors

The findings highlight that contextual factors could substantially influence the associations between PRF and parenting behaviors. In many included studies, the significance and effects of the associations differed between bivariate and multivariate analyses.

The observational setting is an important methodological factor. It can be assumed that PRF is stronger related to parenting behavior measured in emotionally more challenging situations. The context-specific nature of mentalizing ability suggests that PRF would increase with a moderate level of emotional arousal and decrease if the arousal becomes critically stressful (Fonagy and Luyten, 2009). Thus, PRF can be a protective factor in case of moderate child distress or parenting stress. This protective effect was partly supported by one of the included studies (Krink et al., 2018). Especially a direct effect of interactional distress on maternal behavior appears to be related to PRF. This finding highlights the regulatory effect of PRF, particularly because parental sensitivity has shown to be lower in less naturalistic interaction situations (Branger et al., 2019), so that higher parental mental capacity is required. In a moderately distressed situation under less naturalistic conditions, reflective parents would cope better and not be overwhelmed by their own heightened emotions, as other experimental studies have demonstrated (Rutherford et al., 2013, 2015). There is evidence that this is even the case for prenatal PRF (Smaling et al., 2016a). The findings by Huth-Bocks et al. (2014) indicate a more robust association between PRF and maternal behavior in free play situation than teaching task, though the reversed assessment time points for PRF and parenting behavior limit the interpretability of this result. Overall, the findings highlight the importance of observation settings to identify underlying patterns of the association between PRF and parenting quality.

Regarding the socioeconomic environment, only family income was analyzed as a relevant factor. Notably, the interaction effect between paternal PRF and family income was only shown in association with sensitivity to child’s cues and not with social–emotional growth fostering (Buttitta et al., 2019). Sensitivity to child’s cues in the NCAST Teaching Scale measures how parents can structure the task for their children and respond to their children’s interactive cues. Thus, Buttitta et al. (2019) argued that this behavior captures a rather cognitive capacity in the parent–child interaction that might be more affected by socioeconomic hardship than social–emotional growth fostering. Similarly, PRF is related to cognitive capacities such as executive function and can be impaired by chronic stress (Yatziv et al., 2020). There is also evidence of a negative link between PRF and long-term unemployment, which is related to social exclusion and isolation (Sleed et al., 2020). Taken together, PRF can show a protective effect against the negative impact of socioeconomic hardship on specific parenting behavior, while the overlap of cognitive aspects of PRF and specific parenting behavior could particularly interact with the parental socioeconomic environment.

Related to the family environment, Buttitta et al. (2019) showed a predicting effect of maternal PRF on paternal behavior, on which the fathers’ PRF itself did not show a significant effect. This additional finding indicates a complex interplay between maternal and paternal PRF in predicting parenting behavior, which should yet be further investigated (Cooke et al., 2017). For example, recent studies demonstrated the relevance of parents’ RF when reflecting on the couple relationship and the triadic interaction between both parents and the child to be connected with PRF, indicating mutual influences from mothers and fathers (Borelli et al., 2020; León and Olhaberry, 2020).

Further, the cultural background needs to be considered. The difference between two behavioral measures in association with PRF (Dawson et al., 2018) is in line with empirical findings of cultural differences regarding parental mentalization and parent as well as child factors in the assessment of attachment-related behaviors (Dai et al., 2019; Voges et al., 2019). Particularly in collectivistic cultures, the significant meaning of others’ minds and appropriate behavior according to social expectation in parenting context have shown to be different than in individualistic cultures (Aival-Naveh et al., 2019; Lee et al., 2020; Fujita and Hughes, 2021).

### Differences Depending on Sample Types

Sample type is essentially linked with both study settings and other factors regarding the family and social context (Kotchick and Forehand, 2002; Sleed et al., 2020). In the literature of early parenting, at-risk or high-risk samples often involve histories of early adversity or trauma, substance use, psychopathology, and poverty, which are linked with RF impairment (Luyten et al., 2017a; Slade et al., 2019). Especially in early childhood at the age of 0–5 years, the child’s high level of dependency could activate emotional difficulties in parents with their own inner conflicts, leading to lower parenting quality. Although the two direct comparisons between at-risk and comparison groups in this review showed no significant difference (Perry et al., 2015; Suardi et al., 2020), group comparisons might reveal different associations when PRF sub-dimensions are considered. Explicitly testing the effect of psychosocial risks linked with high-risk samples could also be helpful. For example, there is evidence of an indirect effect of psychosocial risks on maternal parenting through prenatal PRF (Smaling et al., 2016a). Besides the effect of financial hardship mentioned above, the effects of psychosocial risks were not investigated directly in the included studies.

As part of psychosocial risks, parental psychopathology is also relevant. Although PRF affects parenting independent of parental psychopathology, impairments in mentalizing ability are linked to most forms of mental disorders (Rostad and Whitaker, 2016; Luyten et al., 2020). Depending on the type and severity of psychopathology, it is likely to be associated with PRF differently. While one of the studies linked pre-mentalizing modes with maternal postpartum depression (*r* = 0.44, *p* = 0.001), another study demonstrated a paradoxical positive link (*r* = 0.47, *p* < 0.01) between higher levels of BPD features and PRF (Krink et al., 2018; Newman-Morris et al., 2020). The latter study also revealed a buffering and protective effect of PRF, moderating the negative impact of distorted maternal representations on maternal non-hostile behavior. Accordingly, another included study hypothesized that PRF might not be directly associated with parenting behavior when severe psychopathology is present (Schechter et al., 2008). Instead, maternal mental representation could be more directly associated with parenting behavior in this context (Schechter et al., 2008; Newman-Morris et al., 2020).

Moreover, in at-risk or high-risk samples, the limited variance and non-normal distribution of the PDI-RF scores are relevant (Sleed et al., 2020). Findings suggest that an effect in the association could be hard to find when study samples show a limited range of PRF scores due to related psychosocial risk factors (Schechter et al., 2008). Low levels of PRF are commonly observed in high-risk samples such as mothers with substance abuse (Hakansson et al., 2018; Adams, 2020), leading to difficulties detecting statistical effects. Nonetheless, maternal adult RF has shown mediating effect on the link between maternal experience of childhood maltreatment and substance use severity (Macfie et al., 2020). Thus, the improvement of PRF might also help high-risk mothers in their self-regulation to process adverse early experiences. It might be meaningful to apply different measures of different types of maternal mentalizing ability to detect this effect.

Finally, maternal and paternal samples should be recognized as having a partially distinct pattern of association between PRF and parenting. Differences between mothers and fathers in levels of PRF and interaction patterns with the infant were also found in other studies (Feldman, 2003; Pajulo et al., 2015; Cooke et al., 2017; Pazzagli et al., 2018). The differences between maternal and paternal samples are consistent with previous studies showing an independent attachment relationship between an infant and each parent (Fonagy and Target, 1997; van IJzendoorn and De Wolff, 1997). The lower PRF level in fathers compared to mothers could be partially linked with differential socialization regarding the gender role, resulting in lower emotional awareness and expression among men (Cooke et al., 2017). Nevertheless, paternal RF shows a unique influence on child development (Benbassat and Priel, 2015). Societal circumstances and gender role expectations (e.g., possibility of paternity leave, role as financial provider) should be considered while investigating paternal PRF and parenting, such as the amount of time spent with the child directly (Brown et al., 2012).

### Limitations

The present review has several limitations. Although the heterogeneity regarding the included sample types was necessary to reveal meaningful differences in the investigated association, this could also include potential confounders related to the sample types. To keep our research question focused, we did not include parental behavior that could not be separated from child behavior, such as synchrony or dyadic attunement. This approach, however, limits the interpretation of our findings since the child’s perspective is also important in this context. Considering the systematic search, besides published peer-reviewed studies, we included two grey literature sources to avoid publication bias. Despite standardized quality assessment, this could also be seen as a limitation. Further, the methodological quality assessment has been adapted to the research context and does not represent a definite rating of the study qualities. Lastly, only two studies were conducted in non-Western countries, and although common in this research area, the sample sizes were mainly small to modest. Hence, the generalizability of our findings is limited.

### Conclusion

Findings of the current review demonstrate that although PRF is generally positively associated with positive parenting and negatively associated with negative parenting, this is not evident for all PRF sub-dimensions depending on sample types (e.g., high-risk vs. community, mothers vs. fathers). Over half of the studies did not compare multiple parenting behaviors concurrently. Especially negative parenting constructs were scarcely examined. The indication of stronger associations in emotionally more challenging interaction situations demonstrates the regulatory effect of PRF on parenting quality under moderate distress. The considerable differences between bivariate and multivariate associations suggest crucial influence from other contextual factors such as socioeconomic status or cultural background. This finding draws attention to consider the family system and the socioeconomic and cultural environment in which parents and their children are situated. For high-risk samples, an investigation of the PRF sub-dimensions is essential. Mixed findings on the role of maternal depression and BPD features highlight the complex interaction between PRF and parental psychopathology. Future research should investigate the factorial models of PRF using various observation settings in diverse sample types (regarding sociodemographic characteristics, psychosocial risk factors, and cultural context) with larger sample sizes using multivariate statistics to generate more insights for embedding PRF into a complex and comprehensive parenting context.

## Data Availability Statement

The original contributions presented in the study are included in the article/Supplementary Material, further inquiries can be directed to the corresponding author.

## Author Contributions

LS developed the research question and study design, conducted the literature search and data extraction, and drafted and revised the manuscript. LS and AG reviewed the studies for eligibility and quality rating. SM and CB contributed to the refinement of the final research question. AG, CB, and SM contributed to the drafting and revision of the manuscript. All authors contributed to the article and approved the submitted version.

## Funding

This work is part of a project supported by the German Psychoanalytic Society (DPG) and the Sigmund-Freud-Foundation (in Frankfurt am Main). The first author is funded by a PhD scholarship from the Hans-Böckler-Foundation (HBS). The research positions of the first and second authors are partially funded by the Jürgen Rickertsen and Georg & Jürgen Rickertsen foundation. The funding sources are not involved in the conduct of this research or the preparation of this article.

## Conflict of Interest

The authors declare that the research was conducted in the absence of any commercial or financial relationships that could be construed as a potential conflict of interest.

## Publisher’s Note

All claims expressed in this article are solely those of the authors and do not necessarily represent those of their affiliated organizations, or those of the publisher, the editors and the reviewers. Any product that may be evaluated in this article, or claim that may be made by its manufacturer, is not guaranteed or endorsed by the publisher.
