# Supplementary material for: Parental Reflective Functioning and Its Association With Parenting Behaviors in Infancy and Early Childhood: A Systematic Review
Source: Front Psychol. 2022 Mar 3;13:765312. doi: 10.3389/fpsyg.2022.765312 (PMC8927808; doi:10.3389/fpsyg.2022.765312)
Supplement: Supplementary file 1 [file Data_Sheet_1.docx]

**Supplementary Material**

**EPHPP QUALITY ASSESSMENT CHECKLIST^[[1]](#footnote-1)^ adapted**

**A) SELECTION BIAS**

**A1 Are the individuals selected to participate in the study likely to be representative of the target population?**

Consider questions from the NICE guideline^[[2]](#footnote-2)^:

- Was the method of selection of participants from the eligible population well described?
- Were the inclusion or exclusion criteria explicit and appropriate?

Criteria: description of sampling method, description of sampling setting, (e.g., in-/exclusion criteria, recruitment sites and procedure), sample size

1. Very likely
2. Somewhat likely
3. Not likely
4. Can’t tell (selection not described)

**A2 What percentage of selected individuals agreed to participate?**

1. 80–100% agreement
2. 60–79% agreement
3. Less than 60% agreement
4. Not applicable*
5. Can’t tell**

*Not applicable: Consider whether it has been described in the paper, why it was not possible to report a response rate.

**Can’t tell: if the response rate was not mentioned AND it’s not possible to calculate the response rate based on reported statistics in the paper.

**RATE SECTION A**

**1 Strong**: The selected individuals are very likely to be representative of the target population (A1 is 1) and there is greater than 80% participation (A2 is 1) or the missing participation rate has been justified well (A2 is 4 with justification).

**2 Moderate**: The selected individuals are at least somewhat likely to be representative of the target population (A1 is 1 or 2) and there is 60–79% participation (A2 is 2). ‘Moderate’ may also be assigned if A1 is 1 or 2 and A2 is 4 (without justification).

**3 Weak**: The selected individuals are not likely to be representative of the target population (A1 is 3); or there is less than 60% participation (A2 is 3) or selection is not described (A1 is 4) and the level of participation is not described (A2 is 5).

**SELECTION BIAS**

**Low** The sample is very likely to be representative of the target population or a specific subgroup. The participation rate is either greater than 80% or well justified for not reporting (e.g., recruitment through flyers or posts on the internet).

**Moderate** The sample is at least somewhat likely to be representative of the target population or a specific subgroup. The participation rate is either between 60–79 % or the recruitment procedure shows indications of justification for not reporting.

**High** The sample is not likely to be representative of the target population or a specific subgroup. The participation rate is less than 60 %.

**B) STUDY DESIGN**

**Indicate the study design**

Ratings adapted using the NICE guideline

Observational designs:

1. cohort study (prospective & retrospective)
2. Case–control studies
3. cross-sectional study
4. Correlation study

Experimental/intervention designs:

1. Randomised controlled trial (RCT)
2. Non-randomised controlled trial (NRCT)
3. Pre–post study aka. Before-and-after (BA) studies

Others:

1. Other specify
2. Can't tell

**RATE SECTION B**

**1 Strong**: articles that described a cohort study, a case-control study, or a RCT.

**2 Moderate**: articles that described a cross-sectional study, a correlational study or a NRCT, a pre-post study.

**3 Weak**: articles that used any other method or did not state the method used.

**E) DATA COLLECTION METHODS**

Only consider the measurement tools on the outcome level (i.e., constructs examined by the review).

Measurement tools must be described as reliable and valid. If ‘face’ validity or ‘content’ validity has been demonstrated, this is acceptable. Some sources from which data may be collected are described below:

- Self-reported data includes data that is collected from participants in the study (e.g., completing a questionnaire, survey, answering questions during an interview, etc.).
- Assessment/Screening includes objective data that is retrieved by the researchers. (e.g., observations by investigators).
- Medical Records/Vital Statistics refers to the types of formal records used for the extraction of the data. Reliability and validity can be reported in the study or in a separate study. For example, some standard assessment tools have known reliability and validity.

**E1 Were data collection tools shown to be valid?**

1. Yes
2. No
3. Can’t tell

**E2 Were data collection tools shown to be reliable?**

1. Yes
2. No
3. Can’t tell

**RATE SECTION E**

**1 Strong**: The data collection tools have been shown to be valid (E1 is 1); and the data collection tools have been shown to be reliable (E2 is 1).

**2 Moderate**: The data collection tools have been shown to be valid (E1 is 1); and the data collection tools have not been shown to be reliable (E2 is 2) or reliability is not described (E2 is 3).

**3 Weak**: The data collection tools have not been shown to be valid (E1 is 2) or validity is not described (E1 is 3) or both reliability and validity are not described (E1 is 3 and E2 is 3).

**DETECTION BIAS**

**Low** Index variable was assessed through a validated and reliable instrument.

**Moderate** Index variable was assessed through a validated instrument which has not been shown to be reliable or its reliability is not described.

**High** The instrument used to assess index variable has not been shown to be valid. Or both reliability and validity are not described.

**F) WITHDRAWALS AND DROP-OUTS**

**F1 Were withdrawals and drop-outs (or missing data in cross-sectional studies) reported in terms of numbers and/or reasons per group?**

1. Yes
2. No

**F2 Indicate the percentage of participants completing the study (or missing data in cross-sectional studies). (If the percentage differs by groups, record the lowest).**

1. 80–100%
2. 60–79%
3. Less than 60%
4. Can’t tell
5. Not Applicable (i.e., Retrospective case-control)

The percentage of participants completing study and the percentage of missing data could also be indicated as reported sample size in the result section.

The combination of F1 = 2 und F2 = 1 was mostly rated in studies that did not report drop-outs or missing values but indicated in results that data from the whole sample has been used. In this case, it also qualifies for a moderate total rating.

**RATE SECTION F**

**1 Strong**: when the follow-up rate is 80% or greater (F1 is 1 and F2 is 1) or missing data was less than 20%.

**2 Moderate**: when the follow-up rate is 60 – 79% (F2 is 2) or missing data was 20–40% or F1 = 2 and F2 = 1 (see comment above).

**3 Weak**: when a follow-up rate is less than 60% (F2 is 3) or if the withdrawals and drop-outs were not described (F1 is 2 or F2 is 4) or missing data was more than 40% or missing data were not described.

**ATTRITION BIAS**

**Low** The follow-up rate is high, or percentage of missing data was low.

**Moderate** The follow-up rate is moderate, or percentage of missing data was tolerable.

**High** The follow-up rate is low, or percentage of missing data was high.

**H) ANALYSES**

**H1 Are the statistical methods appropriate for the study design?**

This refers to the whole statistical analysis of the study (rating on the study level, i.e., not only statistics that has been extracted for the review).

Due to the complexity of statistical analyses and related statistical conditions, this section is only rated based on descriptions of the respective study and common statistical approaches in the relevant research area.

1. Yes
2. No
3. Can’t tell

**H2 Were important potential confounders controlled?**

(e.g., sociodemographic data, obstetric history, psychosocial variables)

1. Yes
2. No
3. Can’t tell

**RATE SECTION H**

**1 Strong**: will be assigned when H1 is 1 and H2 is 1.

**2 Moderate**: will be assigned when H1 is 2 and H2 is 1 OR H1 is 2 and H2 is 2.

**3 Weak**: will be assigned when H1 is 3 and H2 is 2 or 3.

**GLOBAL RATING**

**1 STRONG** (no WEAK ratings in the sections)

**2 MODERATE** (one WEAK rating in the sections)

**3 WEAK** (two or more WEAK ratings in the sections)

1. <https://www.ephpp.ca/quality-assessment-tool-for-quantitative-studies/> [↑](#footnote-ref-1)
2. National Institute for Health and Care Excellence (2012). *Methods for the development of NICE public health guidance* [Online]. Available: https://www.nice.org.uk/process/pmg4 [Accessed January 10, 2019]. [↑](#footnote-ref-2)
