# Supplementary material for: Parental Reflective Functioning and Its Association With Parenting Behaviors in Infancy and Early Childhood: A Systematic Review
Source: Front Psychol. 2022 Mar 3;13:765312. doi: 10.3389/fpsyg.2022.765312 (PMC8927808; doi:10.3389/fpsyg.2022.765312)
Supplement: Supplementary file 2 [file Table_1.docx]

**Supplementary Table 1.** Overview of the included studies.

| First author, year, country | Study design | Sample size | Setting & sample characteristics | Instrument & descriptive data PRF *M* (*SD*) | Instrument & descriptive data parenting behaviour *M* (*SD*) | Statistics used | Associations PRF &  parenting behaviours |
| --- | --- | --- | --- | --- | --- | --- | --- |
| Buttitta, 2019, USA | Cross-sectional | *N* = 77 father-toddler dyads | Community sample, part of a larger study examining mothers’ experiences parenting young toddlers at the Pomona College, California;  toddlers’ age in months *M* (*SD*), range: 23.72 (3.69), 17–31 | PDI-RF (child-focused, adapted);  3.33 (0.67) | NCAST Teaching Scale;  Autonomy support (SEN to cues): 8.90 (1.25)  Social-emotional support (SEGF): 7.45 (1.30) | Pearson  correlation  path analysis  regression-based moderation analysis  regression-based mediation analysis | Child-focused PRF w/   - Autonomy support *r* = .22 (ns.) - Social-emotional support *r* = .04 (ns.);   path child-focused PRF effect on   - Social-emotional support *b* = .50* (*p* = .02)   path child-focused PRF effect on   - Autonomy support *b* = .11 (*p* = .61);   interaction between family income and child-focused PRF effect on   - Social-emotional support *b* = −.15 (*p* = .22)   interaction between family income and child-focused PRF effect on   - Autonomy support Δ*R^2^* = .10, *b* = –.34** (*p* = .008);   child-focused PRF (predictor) effect on   - Social-emotional support (mediator) *b* = .439 (*p* = .07) |
| Dawson, 2018, South Africa | Cross-Sectional | *N* = 50 mother-infant dyads | At-risk sample, part of an evaluation study of a home visiting project in Alexandra Township;  infants’ age in months *M*, range: 4.6, 2.76–15 | PDI-RF;  descriptive data not reported | Ainsworth sensitivity scale & MBQS-mini, using free play & interaction tasks;  descriptive data not reported | Pearson correlation | PRF w/   - Ainsworth SEN *r* = .24 (*p* = .09) - MBQS-mini SEN *r* = .02 (*p* = .91) |
| Dollberg, 2021, Israel | Cross-Sectional | *N* = 68 mother-infant dyads | Community sample, part of a larger cohort study, including women with pregnancy risks;  infants’ age in months *M* (*SD*): 3.84 (0.86) | PDI-RF at 3 months pp;  4.39 (1.36) | CIB (SEN) at 3 months pp;  4.28 (0.49) | Pearson correlation  regression-based mediation analysis | PDI-RF w/ SEN *r* = .14 (ns.)  path PRF (mediator) effect on SEN (outcome) ns. (no coefficients reported) |
| Dunckel, 2003, USA^a^ | Cross-Sectional | *N* = 34 mother-infant dyads | Community sample in New York City;  infants’ age in months range: 4–7.5 | Highpoints/Lowpoints Interview using PDI-RF coding;  4.97 (0.91) | PCIS (Quality of interaction) using free play;  3.84 (0.77) | Pearson correlation | PRF w/ Quality of interaction *r* = .021 (*p* = .91) |
| Ensink, 2019, Canada | Longitudinal | *N* = 88 mother-infant dyads | Community sample in a French-Canadian city | Mini-PRFI at 6 months pp;  4.46 (1.40) | DIP scale (INSEN) at 6 months pp, using interaction task;  2.74 (2.47) | Pearson correlation  hierarchical linear regression  regression-based mediation analysis | PRF w/ INSEN *r* = –.24* (*p* = .03);  PRF effect on INSEN Δ*R^2^* = .02, ß = –.24* (*p* = .04);  path PRF (predictor) effect on INSEN (mediator) *b* = –0.41* |
| Grienenberger, 2005, USA | Longitudinal | *N* = 45 mother-infant dyads | Community sample, first-time mothers, part of a project to explore various aspects of early mother-infant attachment relationships in New York City | PDI-RF at 10 months pp;  descriptive data not reported | AMBIANCE (disrupted affective communication) at 14 months pp, using the Strange situation;  3.33 (1.67) | Pearson correlation | PRF w/ Overall level of disrupted affective communication *r* = –.481*** (*p* < .001) |
| Hasselbeck, 2015, Austria^b^ | Cross-Sectional | *N* = 71 father-child dyads | Community sample, Project "Parenting and co-parenting in infancy";  children’s age in months range: 12–32 | PDI-RF;  3.12 (0.88)  3.6 as “cut-off” | EA Scales (SEN) using picture book situation;  descriptive data not reported | *t* test  path analysis | Higher SEN in high PRF group compared to low PRF group *t*(65) = –3.03**, *d* = 0.78;  path PRF effect on SEN *b* = 0.25 (*p* = .11) |
| Huth-Bocks, 2014, USA | Longitudinal | *N* = 115 mother-infant dyads | Oversampled for mothers w/ childhood maltreatment, Maternal Anxiety during the Childbearing Years project (MACY) | PDI-RF at 16 months pp;  4.40 | MIPCS at 7 months pp, using free play & teaching task;  positive parenting in   - free play: 3.45 - teaching task: 3.21   hostile/intrusive parenting in   - free play: 1.74 - teaching task: 2.08 | Pearson correlation | PRF w/ positive parenting in   - free play *r* = .33***, *r*_part_ = .22* - in teaching task *r* = .21*, *r*_part_ = .13 (ns.)   PRF w/ hostile/intrusive parenting in   - free play *r* = –.28**, *r*_part_ = –.18 (ns.) - teaching task *r* = –.16 (ns.), *r*_part_ = –.06 (ns.)   (partial correlations: controlled for family income risk and maternal age) |
| Krink, 2018, Germany | Cross-Sectional | *N* = 50 mother-infant dyads | Risk sample (mothers w/ pp depression), part of an intervention study at a university medical centre;  infants’ age in month range: 3–10 | PRFQ;  PM: 1.80 (0.73)  CMS: 3.39 (1.22)  IC: 5.87 (0.84) | Mini-MBQS-V using SFP;  0.74 (0.21) in SFP play episode  0.62 (0.31) in SFP re-engagement episode | Pearson correlation | PRF dimension PM w/ SEN in SFP   - play episode *r* = .18 (ns.) - re-engagement episode *r* = –.12 (ns.)   PRF dimension CMS w/ SEN in SFP   - play episode *r* = –.16 (ns.) - re-engagement episode *r* = –.08 (ns.)   PRF dimension IC w/ SEN in SFP   - play episode *r* = –.04 (ns.) - re-engagement episode *r* = –.01 (ns.)   changes in SEN between play and re-engagement episodes w/   - PRF dimension PM *r* = −.24* - PRF dimension CMS *r* = .03 (ns.) - PRF dimension IC *r* = .02 (ns.) |
| Newman-Morris, 2020, Australia | Cross-sectional | *N* = 61 mother-infant dyads | High-risk sample (mothers w/ BPD features) in New South Wales;  infants’ age in month *M* (*SD*): 5.3 (3.2) | PDI-RF;  4.2 (1.3) | EA Scales using  free play;  SEN: 4.5 (1.5)  STRU: 4.0 (1.3)  NON-INTRU: 4.1 (1.4)  NON-HOST: 4.9 (1.6) | Pearson  correlation  regression-based moderation analysis | PDI-RF w/   - SEN *r* = –.12 (ns.) - STRU *r* = –.23 (ns.) - NON-INTRU *r* = –.22 (ns.) - NON-HOST *r* = –.33**;   interaction between PDI-RF and distorted maternal representations effect on NON-HOST Δ*R*^2^ = .058, *b* = .04* (*p* = .027) |
| Perry, 2015, Australia | Longitudinal | *N* = 26 mother-infant dyads:   - *n* = 11 high-risk - *n* = 15 comparison | High-risk group (mothers in opiate substitution treatment) and comparison group w/o risk status | PDI-RF at 6 months pp;  high-risk:   - PDI-RF: 5.00 (1.23)   comparison:   - PDI-RF: 4.67 (1.07) | EA Scales at 6 months pp, using free play;  high risk:   - SEN: 5.60 (.65) - STRU: 5.70 (.57) - NON-INTRU: 6.50 (.00) - NON-HOST: 7.00 (.00)   comparison:   - SEN: 5.33 (3.89) - STRU: 5.21 (1.12) - NON-INTRU: 6.33 (.25) - NON-HOST: 6.92 (.20) | Pearson correlation | Postnatal PRF w/   - SEN *r* = .14 (ns.) - STRU *r* = .00 (ns.) - NON-INTRU *r* = −.12 (ns.) - NON-HOST *r* = −.08 (ns.) |
| Schechter, 2008, USA | Cross-sectional | *N* = 41 mother-toddler dyads | At-risk sample (dyads referred to Infant-Family Service for evaluation);  children’s age in month *M*, range: 32, 8–50 | WMCI-RF (w/ additional probes) at first visit;  3.3 (1.3)  range: 0–5 | AMBIANCE at second visit (1–2 weeks later), using free play & separation–reunion segments;  4.90 (1.43)  range: 1–7 | Linear regression model | PRF effect on Overall level of disrupted affective communication, coefficient not reported (*p* > .4) |
| Sleed, 2013, UK | Pre-Post, intervention evaluation | *N* = 163 mother-infant dyads:   - *n* = 88 IG, - *n* = 75 CG | High-risk sample (mothers in Mother and Baby Unit in prisons);  infants’ age in month *M* (*SD*), range:  4.9 (4.5), 0.2–23.0 in IG  4.4 (4.6), 0.1–18.5 in CG | PDI-RF at baseline & post-treatment;  baseline:   - 3.18 (1.38) in IG - 3.59 (1.47) in CG   post-treatment:   - 3.54 (1.57) in IG - 3.15 (1.33) in CG | CIB (PE) at baseline & post-treatment, using free play;  baseline:   - 19.63 (3.8) in IG - 20.34 (2.9) in CG   post-treatment:   - 19.13 (2.7) in IG - 19.30 (3.2) in CG | Pearson correlation | PRF w/ PE at baseline *r* = .232*  change (baseline to post-treatment) in PRF w/ change in PE *r* = .075 (ns.) |
| Suardi, 2020, Switzerland | Cross-sectional | *N* = 56 mother-toddler dyads:   - *n* = 33 IPV-PTSD - *n* = 75 non-IPV-PTSD | At-risk group (mothers w/ IPV-PTSD) and comparison group (mothers w/o IPV-PTSD) in the metropolitan Geneva area;  toddlers’ age in month *M* (*SD*): 27.5 (9.1) in IPV-PTSD group 26.7 (8.3) in non-IPV-PTSD group | WMCI-RF (with additional probes) at first visit;  4.33 (1.08) in IPV-PTSD group  4.57 (0.66) in non-IPV-PTSD group | CARE-Index using free play at second visit (2–3 weeks later);  IPV-PTSD group   - SEN: 5.06 (1.46) - CONTR: 3.33 (1.71) - UNRESP: 2.91 (1.77)   non-IPV-PTSD group   - SEN: 6.00 (1.04) - CONTR:2.22 (1.24) - UNRESP: 2.30 (1.49) | Pearson correlation  multiple regression analysis | WMCI-RF w/ SEN   - *r* = .36 ** (*p* = .007) in the whole sample - *r* = .34** in IPV-PTSD group - *r* = .33** in non-IPV-PTSD group   WMCI-RF w/ CONTR   - *r* = –.19 (ns.) in the whole sample - *r* = –.23 (ns.) in IPV-PTSD group   WMCI-RF w/ UNRESP   - *r* = –.23 (ns.) in the whole sample - *r* = –.20 (ns.) in IPV-PTSD group;   WMCI-RF effect on SEN β = .33** (*p* = .008) |
| Suchman, 2010, USA | Baseline of an RCT (pilot study) | *N* = 47 mother-child dyads | High-risk sample (mothers with drug use disorders), the Mothers and Toddlers Program (MTP), in a small and ethnically diverse north-eastern city,  children’s age in month: 17.68 (13.82) | PDI-RF;  self-focused: 3.15 (.76)  child-focused: 3.36 (.62) | NCAST Teaching Scale;  SEN to cues: 8.50 (1.39)  CON SEN to cues: 4.39 (.79)  RESP: 8.04 (1.23)  CON RESP: 3.45 (1.04)  SEGF: 7.25 (1.65)  CON SEGF: 1.59 (.87)  CGF: 12.10 (2.04)  CON CGF: 3.77 (1.49) | Multiple linear regression | Self-focused PRF effect on   - SEN to cues *R^2^* = .10, ß = .37* - CON SEN to cues *R^2^* = .07, ß = .31 (ns.) - RESP *R^2^* = .00, ß = .01 (ns.) - CON RESP *R^2^* = .06, ß = –.28 (ns.) - SEGF *R^2^* = .08, ß = .33* - CON SEGF *R^2^* = .05, ß = .27 (ns.) - CGF *R^2^* = .08, ß =.34* - CON CGF *R^2^* = .10, ß = .37*   child-focused PRF effect on   - SEN *R^2^* = .01, ß = –.08 (ns.) - CON SEN *R^2^* = .00, ß = –.07 (ns.) - RESP *R^2^* = .02, ß = –.18 (ns.) - CON RESP *R^2^* = .00, ß = –.01 (ns.) - SEGF *R^2^* = .05, ß = .25 (ns.) - CON SEGF *R^2^* = .05, ß = .25 (ns.) - CGF *R^2^* = .00, ß = –.02 (ns.) - CON CGF *R^2^* = .00, ß = .06 (ns.) |
| Suchman, 2018, USA | RCT | *N* = 84 mother-child dyads | High-risk sample (mothers in the outpatient treatment for substance  abuse), the Mothering from the Inside out (MIO) parenting therapy, at a substance abuse treatment centre located in a small north-eastern city;  children’s age in month *M* (*SD*), range: 27.92 (14.88), 11–60 | PDI-RF at baseline and post-treatment;  baseline   - self-focused: 2.94 (0.65) - child-focused: 3.18 (0.59)   post-treatment   - self-focused: 2.98 (0.61) - child-focused: 3.32 (0.55) | CIB (SEN) at baseline, post-treatment and 3-month follow-up, using Curiosity Box Paradigm;  baseline: 3.50 (0.73)  post-treatment: 3.49 (0.78)  3-month follow-up: 3.44 (0.76) | Hierarchical regression analysis | effect on change in SEN at 3-month follow-up:   - change (baseline to post-treatment) in self-focused PRF *R^2^* = 0.00, ß = 0.01 (significance & *p*-value not reported) - change in child-focused PRF *R^2^* = 0.02, ß = 0.14 (significance & *p*-value not reported) |
| *Note*. ^a^ Doctoral dissertation. ^b^ Master’s thesis.  w/ = with; w/o = without; ns. = not significant (if *p*-value not reported); pp = postpartum; vs. = versus; IG = intervention group; CG = control group; RCT = randomized controlled trial; BPD = borderline personality disorder; IPV-PTSD = interpersonal violence-related posttraumatic stress disorder; PDI-RF = Parent Development Interview-revised with reflective functioning coding; PRF = parental reflective functioning; Mini-PRFI = Mini-Parent Reflective Functioning Interview; PRFQ = Parental Reflective Functioning Questionnaire; PM = Pre-mentalizing modes; CMS = Certainty about mental states; IC = Interest and curiosity in mental states; WMCI-RF = Working Model of the Child Interview with reflective functioning coding; NCAST = Nursing Child Assessment Satellite Training; MBQS/-V = Maternal Behavior Q-sort/revised; PCIS = Parent/Caregiver Involvement Scale; DIP = Disconnected and Extremely Insensitive Parenting; AMBIANCE = Atypical Maternal Behavioral Instrument for Assessment and Classification; CIB = Coding Interactive Behavior; EA = Emotional Availability; MIPCS = MACY Infant–Parent Coding System; CARE-Index = Child-Adult Relationship Experimental Index; SFP = Still-Face-Paradigm; SEN = Sensitivity; SEGF = Social-emotional growth fostering; INSEN = Insensitivity; STRU = Structuring; NON-INTRU = Non-intrusiveness; NON-HOST = Non-hostility; PE = Positive engagement; INTRU = Intrusiveness; CONTR = Controlling; UNRESP = Unresponsive; RESP = Response to distress; CON = Contingency; CGF = Cognitive growth fostering. * *p* < .05; ** *p* < .01; *** *p* < .001. *p*-value specified in the table if reported. | | | | | | | |
